# Polar rotor scattering as atomic-level origin of low mobility and thermal conductivity of perovskite CH_3_NH_3_PbI_3_

**DOI:** 10.1038/ncomms16086

**Published:** 2017-06-30

**Authors:** Bing Li, Yukinobu Kawakita, Yucheng Liu, Mingchao Wang, Masato Matsuura, Kaoru Shibata, Seiko Ohira-Kawamura, Takeshi Yamada, Shangchao Lin, Kenji Nakajima, Shengzhong (Frank) Liu

**Affiliations:** 1 Neutron Science Section, Japan Proton Accelerator Research Complex, Japan Atomic Energy Agency, Tokai, Ibaraki 319-1195, Japan; 2 Key Laboratory of Applied Surface and Colloid Chemistry, National Ministry of Education, Institute for Advanced Energy Materials, School of Materials Science and Engineering, Shaanxi Normal University, Xi’an 710119, China; 3 Department of Mechanical Engineering, Materials Science and Engineering Program, FAMU-FSU College of Engineering, Florida State University, Tallahassee, Florida 32310, USA; 4 Neutron Science and Technology Center, Comprehensive Research Organization for Science and Society, Tokai, Ibaraki 319-1106, Japan; 5 Dalian Institute of Chemical Physics, Dalian National Laboratory for Clean Energy, Chinese Academy of Sciences, Dalian 116023, China

## Abstract

Perovskite CH_3_NH_3_PbI_3_ exhibits outstanding photovoltaic performances, but the understanding of the atomic motions remains inadequate even though they take a fundamental role in transport properties. Here, we present a complete atomic dynamic picture consisting of molecular jumping rotational modes and phonons, which is established by carrying out high-resolution time-of-flight quasi-elastic and inelastic neutron scattering measurements in a wide energy window ranging from 0.0036 to 54 meV on a large single crystal sample, respectively. The ultrafast orientational disorder of molecular dipoles, activated at ∼165 K, acts as an additional scattering source for optical phonons as well as for charge carriers. It is revealed that acoustic phonons dominate the thermal transport, rather than optical phonons due to sub-picosecond lifetimes. These microscopic insights provide a solid standing point, on which perovskite solar cells can be understood more accurately and their performances are perhaps further optimized.

## Abstract (web summary)

Clarifying the atomistic behaviour of materials will lead to a deeper fundamental understanding and the rational design of future materials. Using time-of-flight quasi-elastic and inelastic neutron scattering measurements Li *et al*. study the atomic motions of metal-halide perovskite CH_3_NH_3_PbI_3_.

Over last few years, the inorganic–organic hybrid perovskite solar cells have taken the worldwide research community by storm^1^,^2^,^3^.Typically, the energy conversion efficiencies of solar cells based on methylammonium lead iodide (CH_3_NH_3_PbI_3_) have been improved from the starting 4% in 2009 to higher than 20% in 2015 (refs 4, 5). Very recently, the successes in growth of inch-sized high-quality single crystals and the device integration on wafers have paved the route to large-scale practical photovoltaic applications^6^,^7^. These hybrid compounds exhibit distinct physical properties from conventional semiconductors. Their hot-phonon bottleneck effect of energetic carriers is obviously stronger than that of GaAs and other inorganic perovskites, which has been attributed to the acoustic-optical phonon up-conversion^8^,^9^,^10^. The mobility of charge carriers is relatively smaller compared with classical semiconductors^1^,^2^,^3^,^11^,^12^ and the scattering mechanism is still under debate^13^,^14^,^15^. Resembling the charge transport, the thermal transport is unusual as well. The thermal conductivity is surprisingly low, ∼0.5 Wm^−1^K^−1^ for single crystals at room temperature^16^, which is tenfold lower than that of GaAs (ref. 12) and is even lower than that of amorphous silicon^17^.

It is evident that atomic dynamics underlies these peculiarities. However, the atomic-level description of CH_3_NH_3_PbI_3_ is complicated by the hybrid nature where both molecular jumping rotations and phonon excitations have to be taken into account. Moreover, these two components also interact via hydrogen bonds between H and I (refs 18, 19, 20). The best approach for this issue is, without a doubt, inelastic neutron scattering (INS). The density functional theory (DFT) lattice dynamics calculations indicate that low-energy phonons are entirely composed of motions of Pb and I (refs 21, 22, 23, 24, 25, 26), which are easily excited at relatively lower temperatures and hence take main parts in determining the physical properties. These phonons can be efficiently probed throughout the Brillouin zones, due to the larger coherent scattering cross-sections of Pb and I. Simultaneously, the incoherent scattering cross-section of H assures the individual motions of molecules can be determined by extracting the quasi-elastic broadening underneath the elastic line, which is known as quasi-elastic neutron scattering (QENS) method^27^. In this work, we show the complete phonons and jumping rotational modes across the low-temperature phase transition, which are obtained by measuring a large single crystal at two high-resolution time-of-flight neutron spectrometers that cover a wide energy window ranging from 0.0036 to 54 meV. These results are well supported by the complementary molecular dynamics (MD) simulations. It is revealed that the molecular dipole order plays a dominant role in determining charge transport and thermal transport properties of CH_3_NH_3_PbI_3_.

## Results

### Jumping rotational modes of [CH_3_NH_3_]^+^

**Figure 1:**
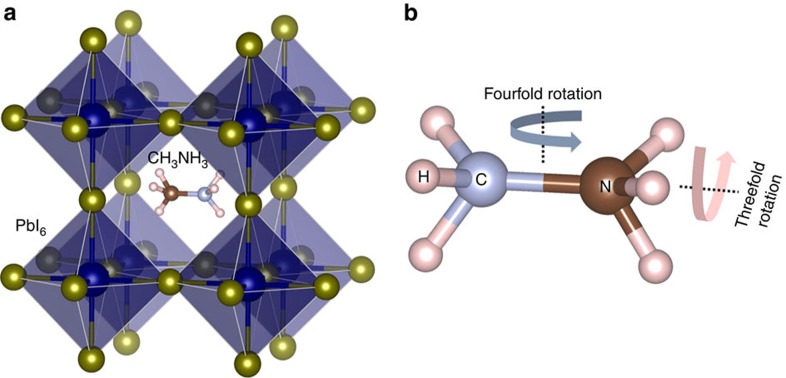
Crystal structure of CH_3_NH_3_PbI_3_. (**a**) The schematic diagram of the perovskite structural unit. The organic cation is located in the centre of PbI_6_ octahedral cage. The real structures are complex due to the orientational disorder of organic cations. (**b**) Geometry of the molecule with schematic drawing of the threefold and fourfold jumping rotational modes.

CH_3_NH_3_PbI_3_ crystallizes in the common perovskite structure where the organic cation [CH_3_NH_3_]^+^ occupies the centre of the PbI_6_ octahedral cage, as shown in Fig. 1a. Neutron and X-ray powder diffraction investigations suggest that it undergoes successive phase transitions, from cubic (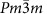) to tetragonal (*I*4/*mcm*) at 330 K, and then to orthorhombic (*Pnma*) at 165 K (refs 28, 29). Our single crystal exhibits a sharp phase transition between 160 and 165 K, as shown in Supplementary Fig. 2, which is in agreement with these reports. While it is believed that this transition is coupled with orientation of [CH_3_NH_3_]^+^, current reports on rotational dynamics are contradictory^30^,^31^, probably owing to the lack of results on single crystals. Given that this compound is extremely sensitive to ambient conditions^32^,^33^, measurements on a single crystal (with minimized surface contribution) are anticipated to deliver more intrinsic information. Here, the accurate jumping rotational dynamics of molecules is obtained by performing ultrahigh energy resolution QENS measurements on the large single crystal at the backscattering neutron spectrometer, DNA.

**Figure 2:**
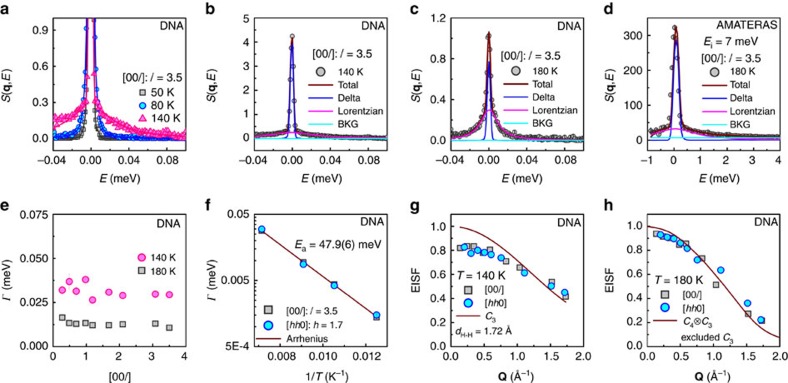
Jumping rotational dynamics of molecules in CH_3_NH_3_PbI_3_. (**a**) *S*(**q**, *E*) at *l*=3.5 along [00*l*] at 50, 80 and 140 K obtained at DNA. (**b**) The spectral fitting of *S*(**q**, *E*) at 140 K for *l*=3.5 along [00*l*] direction, by including a delta function, a Lorentzian function and a constant background (BKG). (**c**,**d**) The spectral fitting of *S*(**q**, *E*) obtained at DNA and at AMATERAS at *l*=3.5 along [00*l*] direction at 180 K, respectively. (**e**) The half width at half maximum (*Γ*) of the Lorentzian components derived in the fitting, as a function of momentum transfer at 140 and 180 K. (**f**) The temperature dependence of *Γ* for the orthorhombic phase, fitted to Arrhenius relation 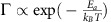, where *E*_a_ is the activation energy of the jumping rotational mode and *k*_B_ is Boltzmann constant. (**g**,**h**) the EISF at 140 and 180 K compared with *C*_3_ model and *C*_4_⨂*C*_3_ model, respectively. In the latter, the *C*_3_ component is excluded. To compare two directions, the real momentum transfer **Q** is used.

First, we demonstrate the jumping rotational dynamics in the orthorhombic phase. As shown in Fig. 2a, the spectrum at 50 K is basically described by a delta function convoluted to the instrumental resolution (the full width at half maximum is ∼0.0036, meV). Around 80 K, the QENS signal described by a Lorentzian function is identified underneath the elastic line, indicative of the activation of rotations. The QENS intensity becomes more noticeable at 140 K. The momentum-transfer averaged spectra along [00*l*] direction are systematically fitted by including a Lorentzian function, a delta function and a constant background, which are convoluted to the instrumental resolution. An example is given at 140 K in Fig. 2b. The half width at half maximum of the Lorentzian function, *Γ*, is examined as a function of momentum transfer and temperature. As shown in Fig. 2e, *Γ* is almost independent on *l*. Since *Γ* is inversely related to the relaxation time, such a value gives rise to an average relaxation time of 23(1) ps at 140 K. The temperature dependence is fitted to the Arrhenius relation and the activation energy ∼47.9(6) meV is obtained (Fig. 2f). The elastic incoherent structure factor (EISF), defined as the ratio between elastic intensity and the sum of QENS and elastic intensity^27^, is shown in Fig. 2g at 140 K. It is described by the threefold jumping rotational (*C*_3_) model, in which 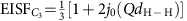
27, where *j*_0_ is the zeroth-order spherical Bessel function and *d*_H–H_ is the average distance between two adjacent protons, ∼1.72 Å in terms of neutron powder diffraction results^28^ (see Supplementary Fig. 3 for details).

In the tetragonal phase, the jumping rotational dynamics becomes more complicated. The spectrum obtained at 180 K at DNA displays a narrower QENS profile than that at 140 K, as compared in Fig. 2b,c. The relaxation time is determined to be 64(2) ps. At the same time, a much broader Lorentzian function is found by using the cold neutron time-of-flight spectrometer AMATERAS, which provides a much wider energy window (Fig. 2d). Such a large width corresponds to a relaxation time as short as 0.71(3) ps. Likewise, EISF is calculated based on DNA data by following the same procedure as above^27^. However, the derived EISF is overestimated because QENS intensity of the faster mode is missed in the total intensity. The recent group theory analysis on jumping rotational dynamics of CH_3_NH_3_PbI_3_ suggests a coupled *C*_3_ and fourfold (*C*_4_) mode in the tetragonal phase^31^. Therefore, we compare the derived EISF with this *C*_4_⨂*C*_3_ model. Shown in Fig. 2h is its comparison with the ratio between elastic intensity and total intensity excluded the *C*_3_ component. The detailed calculation is given in Supplementary Fig. 4. It can be seen that the experimental data is well reproduced in this way, indicating that a *C*_4_ mode sets in after the phase transition to the tetragonal phase and the *C*_3_ mode becomes ∼30 times faster. Hereto, we are able to conclude on the jumping rotational dynamics. In the orthorhombic phase, the protons in CH_3_ and NH_3_ undergo jumping rotations with respect to the C–N axis. The relaxation time is dramatically shortened in the tetragonal phase while the C–N axis starts to rotate with respect to the *c* axis of the crystal structure. The rotational modes are illustrated in Fig. 1b. Our results about the jumping rotational dynamics are in agreement with the recent QENS measurements on a powder sample by Chen *et al*.^31^, while are different from those by Leguy *et al*.^30^ in which freezing of modes is not detected. The relaxation time of the *C*_4_ mode we determined is very close to that identified in previous MD simulations, ∼40 ps (ref. 34).

### Phonons of the orthorhombic and tetragonal phases

**Figure 3:**
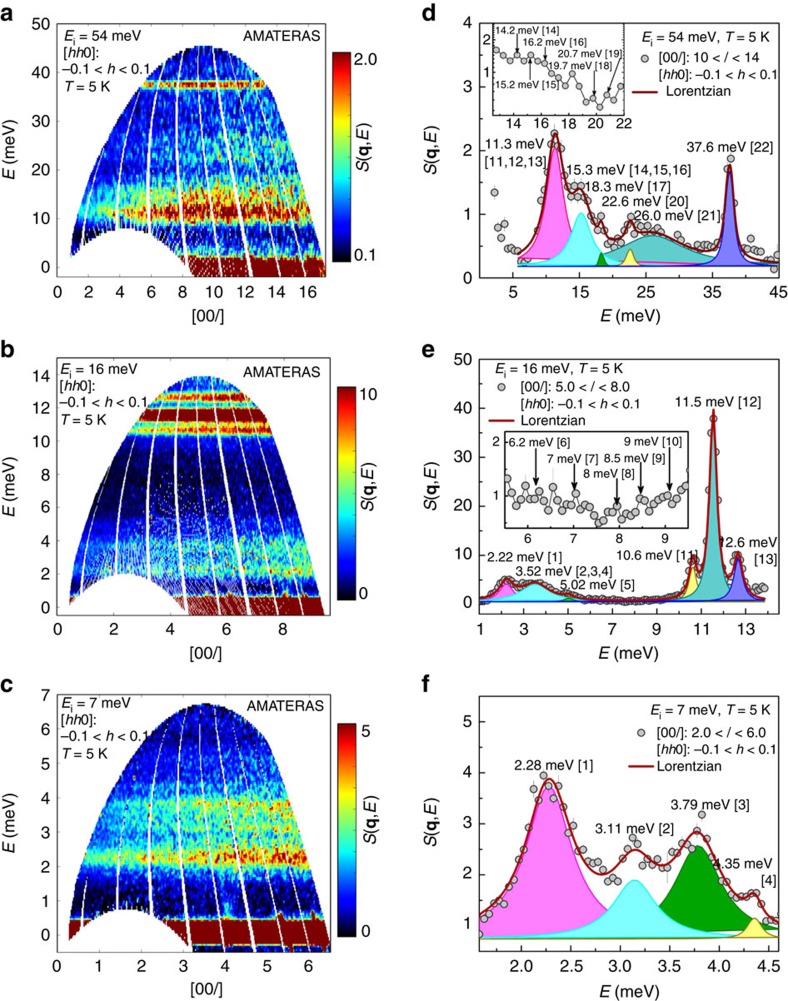
Longitudinal phonon spectra at 5 K along [00*l*] obtained at AMATERAS. (**a**–**c**) The contour plots of *S*(**q**, *E*) obtained with *E*_i_ of 54, 16 and 7 meV. (**d**–**f**) Momentum-transfer averaged spectra and multiple Lorentzian-function fitting to highlight the energies of modes. The peak position for each component is labelled. The insets highlight small shoulders or humps overlooked by the fitting. For the case of [*hh*0], please refer to Supplementary Fig. 5.

The jumping rotational dynamics of an individual molecule is well understood above and now we move to the collective dynamics, that is, phonons, which are measured by selecting several incident energy of neutrons (*E*_i_) at AMATERAS. The obtained dynamic structure factor *S*(**q**, *E*) is shown in Fig. 3 at 5 K in the direction of [00*l*]. With *E*_i_ of 54 meV, the full view of *S*(**q**, *E*) is provided. The momentum-transfer averaged spectrum at 10 < *l* < 14 (Fig. 3d) indicates six prominent modes located at 11.3, 15.3, 18.3, 22.6, 26.0 and 37.6 meV, respectively. Except them, there are several less obvious shoulders or humps, as arrowed in the inset. With smaller *E*_i_, more details are revealed. We find the peak at 11.3 meV shown in Fig. 3d is actually a superposition of three peaks at 10.6, 11.5 and 12.6 meV (Fig. 3b,e). There seems a gap located between 6 and 10 meV, but several weaker modes are roughly identified as highlighted in the inset. The data with *E*_i_ of 7 meV (Fig. 3c,f) suggests four low-energy modes at 2.28, 3.11, 3.79 and 4.35 meV. Thanks to the high-resolution, we are able to identify several modes that are overlooked in the previous Raman scattering^26^ and INS measurements^21^. Particularly, the latter and the associated DFT calculations are compared with our experimental results and MD simulations, as listed in Table 1. The numbers correspond to ones appearing in the square brackets of the labels in Fig. 3d–f and Supplementary Fig. 6. It can be seen that our measurements match well with the simulations, except the modes 21 and 22, which are dominated by the molecular twist motion^24^. This discrepancy is also reported in another MD simulation study using the same potential functions^24^, and might be related to the zero-point-energy fluctuations that lead to more lattice distortions as suggested in the DFT calculations on *P*1 symmetry^21^.

**Figure 4:**
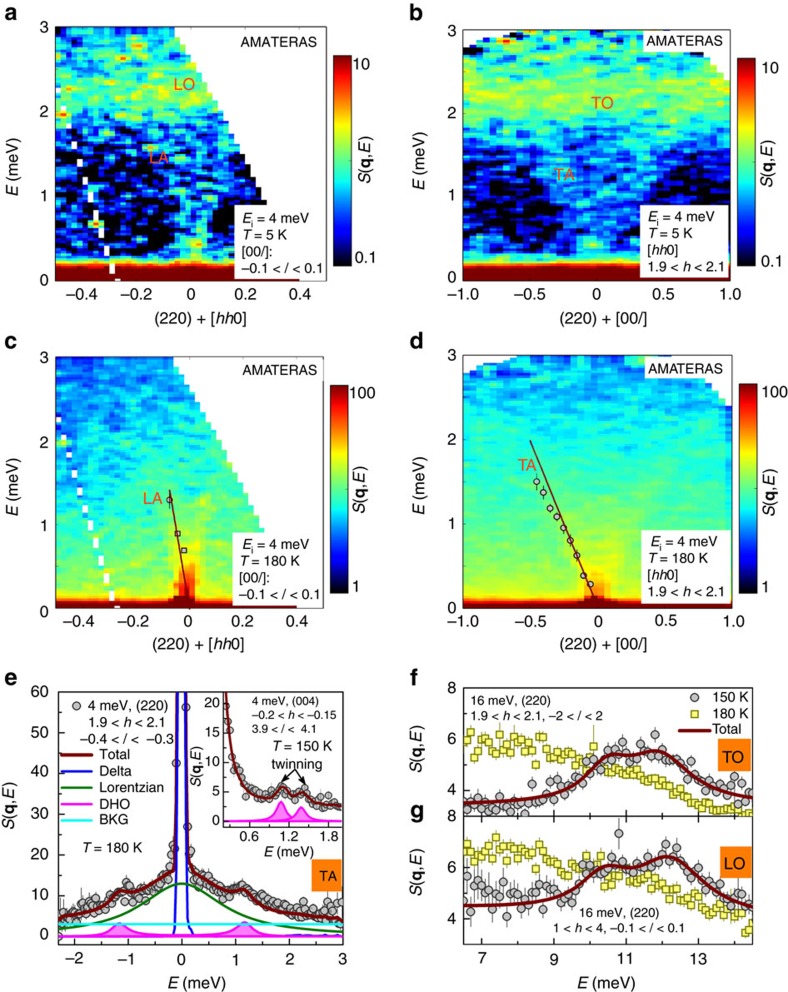
Phonon broadening at the orthorhombic-to-tetragonal phase transition. (**a**,**c**) Longitudinal phonons at 5 and 180 K of the (220) Brillouin zone; (**b**,**d**) Transverse phonons at 5 and 180 K of the (220) Brillouin zone. TA, TO, LA and LO phonons are labelled. The filled circles and square in **c** and **d** are dispersions determined by fitting energy-transfer averaged and momentum-transfer averaged spectra, respectively. The solid lines represent the slopes as approaching the zone centre, which characterize the group velocities. (**e**) Fitting of momentum-transfer averaged TA phonon spectrum of the (220) Brillouin zone at 180 K. The Lorentzian and damped harmonic oscillator (DHO) functions describe the quasi-elastic component and the TA phonon, respectively. The inset shows the TA phonon at 150 K of the (004) Brillouin zone. The presence of two peaks is because the crystal is twinned in the orthorhombic phase (also see Supplementary Fig. 11). (**f**) Fitting of momentum-transfer averaged TO and (**g**) LO phonons spectra of the (220) Brillouin zone at 150 and 180 K with *E*_i_ of 16 meV.

Detailed acoustic phonons of the Brillouin zone (220) are examined with *E*_i_ of 4 meV. Shown in Fig. 4a–d are the longitudinal and transverse phonons at 5 and 180 K, respectively. Even though the background level is high, the dispersions can be seen in both directions. At 5 K, the dispersion curve of longitudinal acoustic (LA) phonon emanates from zone centre of (220), undergoes a steep increase and finally approaches the top in the middle of zone centre and zone boundary (*h* about 0.2), where it is overlapped with longitudinal optical (LO) phonons. The dispersion of transverse acoustic (TA) phonon exhibits a moderate slope. Intense transverse optical (TO) phonons are also present. At 180 K, the intensity of phonons is fairly enhanced and we determine the accurate dispersions of LA and TA phonons by fitting the momentum-transfer (filled circle) or energy-transfer (filled square) averaged spectra. The solid lines represent the slopes of dispersion curves near the zone centres, which determine the group velocities: *ν*_LA_=2841, m s^−1^ and *ν*_TA_=1155, m s^−1^. Shown in Fig. 4e is the fitting of the TA phonon, which yields the lifetime of 3.61(42) ps in the middle of the zone boundary and zone centre. For the LA case, we determine that the lifetime is ∼4.39(46) ps at 180 K in the vicinity of the zone centre (Supplementary Fig. 7). Recently, Whalley *et al*.^35^ have calculated the lifetimes by considering the phonon–phonon interaction. At 300 K, the lifetimes of phonons range around 2 ps, which is very close to our results. The product of lifetime and group velocity defines mean-free-path. At 180 K, short lifetimes and small velocities yield mean-free-paths of ∼125 and 42 Å for the LA phonon and TA phonon, respectively. Since we have first determined these critical quantities, it is allowed to compare with previous theoretical counterparts. Listed in Table 2 is the comparison of velocities and mean-free-paths of acoustic phonons^35^,^36^,^37^,^38^. It is worth noting that our INS results are well supported by the MD simulations based on the same potential functions as we used here^34^,^38^.

More strikingly, the LO and TO phonons around 2.28 meV completely vanish at 180 K. The data of the (004) zone (Supplementary Fig. 8) indicates that they survive at 150 K. Thus, we compare the optical phonons located at 10.7 and 12.6 meV at 5, 150 and 180 K, which are two of strongest optical phonons observed in the spectra. At 5 K, these optical phonons are well-defined, as shown in Fig. 3e. With warming up to 150 K, the lifetimes of TO phonons (Fig. 4f) at 10.7 and 12.6 meV are reduced down to 0.91(15) and 0.62(7) ps while those of LO phonons (Fig. 4g) are reduced down to 0.82(5) and 0.57(2) ps, respectively. These lifetimes are much shorter than 5.40(88) ps for the TA phonon (inset of Fig. 4e). At 180 K, just above the phase transition, both TO and LO phonons are too broadened to be distinguished. This means the actual lifetimes at 180 K are much shorter than those at 150 K. This dramatic broadening is greatly consistent with the Raman scattering study in which the lifetime is shortened from 0.6 to 0.15 ps (ref. 26). The characteristic timescales of jumping rotational modes and phonons are summarized in Table 3.

### MD simulations of phonons through the phase transition

**Figure 5:**
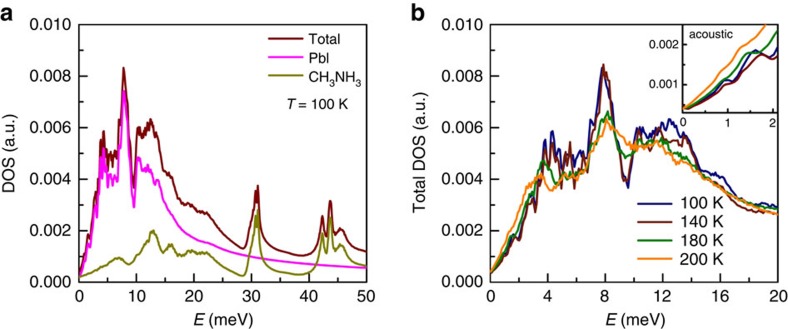
Phonon DOS calculated in simulations. (**a**) The total and partial (contributed by the inorganic and molecular species) DOS at 100 K. (**b**) The total DOS at 100, 140, 180 and 200 K. The inset shows the acoustic phonons located in the low-energy region, which are less broadened than the optical ones during the orthorhombic-to-tetragonal phase transition.

The finite-temperature phonon excitations are considered in classical MD simulations using recently developed MYP potential functions^34^. In Fig. 5a, we show the phonon density of state (DOS) at 100 K. It is clear that motions of the inorganic part are dominant in the low-energy region while the high-energy spectrum is mostly contributed by molecular motions. The energy of each mode is determined in Supplementary Fig. 6 and compared with the experimental counterpart in Table 1. The distinct changes of lifetimes of acoustic and optical phonons through the phase transition are consistent with the simulations where the DOS spectrum of optical phonons is much more broadened than that of acoustic ones. As shown in Fig. 5b, the peaks of optical phonons (between 2 and 16 meV) are remarkably broadened in the tetragonal phase, in agreement with the broadening shown in Fig. 4f,g (for medium-energy optical phonons) and Supplementary Fig. 8 (for low-energy optical phonons). Instead, acoustic phonons shown in the inset are still distinguishable even if their intensities are much weaker than those of optical phonons. The significantly reduced lifetimes of optical phonons in the tetragonal phase imply an additional decay channel taking effect except the phonon–phonon interaction. Whalley *et al*.^35^ have considered the phonon–phonon interaction and directly calculate the lifetimes. It is shown that there are similar lifetimes for phonons with frequencies below ∼3.5 THz (∼14 meV). Namely, the phonon decay based on anharmonicity in this energy region is not selective. In contrast, the MD simulations including the long-range electrostatic interactions indicate that optical phonons are more broadened than acoustic ones. Note that the experimentally observed dipole order has been reproduced using the same methodology of simulations^34^. Thus, the additional scattering is most likely attributed to the activated orientational disorder of dipoles in the tetragonal phase, which is similar to the phonon scattering by paraelectric centres^39^,^40^.

## Discussion

In the tetragonal phase, the temperature dependence of mobility shows a −3/2 power-law, which was initially attributed to the scattering by acoustic phonons^13^. Later, both the experimental investigation of photoluminescence spectra and the transport theoretical study manifest the Fröhlich interaction between charge carriers and LO phonons is the major scattering mechanism^14^,^15^. Indeed, considering the deformation potential and the piezoelectric potential as major mechanisms, the calculated mobility is much larger than experimentally measured one^41^,^42^, indicating that acoustic phonons are less important. Notwithstanding, it is still unclear why the mobility of hybrid perovskites is quite small compared with other inorganic semiconductors that have comparable Fröhlich coupling strengths^14^. More importantly, such a scenario is not able to reproduce the critical-like temperature dependence of mobility at the orthorhombic-to-tetragonal phase transition^43^.

According to the current structural model, the orthorhombic phase is antiferroelectric^28^. Our QENS data suggests that the dipoles are disordered in the picosecond temporal scale in the tetragonal phase. Thus, this orthorhombic-to-tetragonal phase transition is indeed an order-to-disorder transition of dipoles, just as the dielectric constant displays a sharp peak at the transition temperature^44^. Namely, CH_3_NH_3_PbI_3_ is not only a polar semiconductor like GaAs, but it is also antiferroelectric. Extensive earlier reports on electrical transport properties of ferroelectric perovskite oxides and IV–VI semiconductors indicate the critical-point polarization fluctuations result in a singularity of resistivity at the transition temperatures, that is, a positive resistivity-temperature slope as approaching the transition temperature from the low-temperature side^45^,^46^,^47^. Similarly, the dipole scattering is also active in hybrid perovskites with polar cations. The important role of molecular dipoles is manifested at the sharp difference of the magnitude of mobility of hybrid CH_3_NH_3_PbBr_3_ and all-inorganic CsPbBr_3_ (ref. 43). At ∼120 K where both compounds crystallize in orthorhombic structures, the mobility of the former is about 5 times lower. In addition, the critical anomaly has been analytically solved for antiferroelectrics, given as 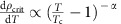
at *T*>*T*_c_ and as 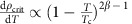
at *T*<*T*_c_, where *ρ*_crit_ is critical resistivity, *T*_c_ is transition temperature, *α* and *β* are critical exponents^47^. Thus, one obtains 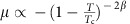
at *T*<*T*_c_. We find the temperature dependence of mobility near the phase transition of CH_3_NH_3_PbI_3_ can be well reproduced with an extremely small value of *β* (Supplementary Fig. 9), indicative of the first-order nature^48^. The sharp change of mobility at the phase transition well coincides with the activation of dipole disorder (see Table 3).

Even though the molecular dipoles are disordered on atomic level as they undergo ultrafast *C*_4_ jumping rotational mode in the picosecond temporal scale, it is possible that there are nanoscale/mesoscale correlations with slower dynamics^49^. This kind of large-scale structures are compatible with ferroelectric domains predicted in DFT^50^ and observed using piezoelectric force microscopy^51^. They are perhaps relevant to the polaron picture proposed for hybrid perovskites^52^, which is the key to understand the extremely low recombination rate of carriers.

It is known that lattice thermal conductivity is equal to 1/3*Cv*^2^τ where *C* is the lattice specific heat, τ is the average group velocity and τ is the average lifetime of phonons^53^. Even through the origin of ultralow thermal conductivity is naturally ascribed to either lower bulk modulus (proportional to *v*^2^) or shorter τ, the profound mechanism is still unclear due to the lack of the complete phonon data, especially acoustic phonons. Our INS study suggests the contribution of optical phonons to thermal transport is marginal because of the significantly shortened lifetimes. On the contrary, the thermal transport is dominated by acoustic phonons. Their nanoscale mean-free-paths together with smaller velocities are responsible for such a small thermal conductivity. Experimentally, the thermal conductivity exhibits a smooth temperature dependence except an abrupt discontinuity at the transition owing to the anomaly of the specific heat^16^ (Table 3). This is well consistent with the less varied lifetimes of acoustic phonons across the phase transition. If the optical phonons were major heat carriers, a dramatic drop of thermal conductivity would be expected at the transition. Molecular vibrations have been recognized to be detrimental to thermal transport^35^,^54^,^55^. The anharmonic DFT calculations by Whalley *et al*.^35^ and MD simulations with MYP potential functions by Caddeo *et al*.^54^ suggest the scattering of low-energy phonons (mostly dominated by the inorganic part) by molecular vibrations acts as the major decay channel, but in another simulation by Hata *et al*.^55^ molecular vibrations only mediate the interaction between phonons consisting of PbI motions. Our results seem to agree with the first case because the twist mode at 37.6 meV is significantly broadened even in the orthorhombic phase (Supplementary Fig. 10).

Given that the molecular vibrations (high-energy optical phonons) are very flat modes, their interaction with acoustic phonons is in analogy to the rattling-mode-phonon coupling in the paradigm of electron-crystal phonon-glass^56^, where less dispersive modes highly scatter acoustic phonons, leading to glassy-like thermal conductivity^57^. It is considerably promising to achieve superior thermoelectric materials in hybrid inorganic–organic perovskites by enhancing charge transport. Simulations indicate the thermoelectric figure-of-merit of CH_3_NH_3_PbI_3_ can reach to 3 at 600 K with optimal carrier density of 10^19^ cm^−3^ (ref. 58). It is also predicted that CH_3_NH_3_SnI_3_ may have much higher value of figure-of-merit than Pb case^59^. Moreover, the low thermal conductivity does not only directly enable hybrid perovskites to be appealing thermoelectric materials, but also plays a fundamentally important part in photovoltaic properties. The suppressed thermal conduction promotes the acoustic-optical phonon up-conversion, benefitting the slow cooling rate of energetic carriers^10^. Such an enhanced phonon bottleneck effect facilitates the realization of the hot-carrier solar cell concept with an extremely high power conversion efficiency^60^.

In summary, we have precisely determined jumping rotational dynamics of molecules and phonons in hybrid perovskite CH_3_NH_3_PbI_3_ by conducting high-resolution QENS and INS measurements, which are all consistent with the finite-temperature MD simulations. The characteristic timescales of various dynamics are completely revealed. The dipole order of organic cations plays a decisive role in determining the physical properties such as carrier mobility and thermal conduction, distinguishing from inorganic perovskites. Such observations are probably general for all hybrid compounds with polar molecules. These fundamental insights are beneficial to further study of perovskite solar cells.

## Methods

### Single crystal growth and mounting

The crystal was grown by using the solution method described in ref. 6. The single crystal used in neutron measurements is 2.32 g in weight and well-faceted. The as-grown single crystal was sealed into a plastic package under vacuum for storage and transportation. The crystal was mounted onto an aluminium holder in a glove box under helium atmosphere. They were sealed into an aluminium can using indium wire in the glove box. The whole process was completed without exposure to air to minimize the contamination by humidity and oxygen. The details of the sample are given in Supplementary Information, including Supplementary Figs 1, 11 and 12.

### QENS and INS measurements

The high-resolution QENS and INS measurements were performed at the inverse-geometry time-of-flight chopper spectrometer BL02 DNA with *E*_i_=2.084 meV and at the direct-geometry time-of-flight chopper spectrometer BL14 AMATERAS with *E*_i_ of 54, 16, 7 and 4 meV of J-PARC in Japan^61^,^62^. The instrumental resolutions are summarized in Supplementary Table 1. The crystal was aligned at room temperature at DNA. It took 12 h to cool down to 140 K from room temperature at which it crystallizes in the tetragonal structure. At base temperature of 10 K, the width of the elastic peak is ∼0.0036, meV, which is equal to the instrumental resolution of DNA. The crystal is twined in the orthorhombic phase, as shown in Fig. 4e and Supplementary Fig. 11. The four-dimension *S*(q, *E*) data were reduced and visualized by using Utsusemi suite^63^, along [*hh*0], [00*l*] and [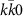] based on a tetragonal unit cell (*a*=8.80625 Å, *c*=12.712 Å). The component along [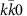] is limited by setting −0.1<*k*<0.1. The one-dimension spectra were fitted in PAN of DAVE^64^.

### MD simulations

MD simulations were performed using the massively parallelized LAMMPS package^65^ and the recently developed classical MYP force field^34^, which reasonably reproduces the physical properties of CH_3_NH_3_PbI_3_ (refs 38, 66, 67). In such a force field, non-bonded pairwise interactions are described by the sum of Buckingham, Lennard-Jones and long-range electrostatic potentials. Intra- and inter-molecular interactions of organic cations CH_3_NH_3_^+^ are described by the AMBER force field^68^. The cut-off distances for all the interactions are chosen to be 10 Å. A time step of 0.5 fs was used in all simulations. After energy minimization, simulation models were first equilibrated for 250 ps under the *NPT* ensemble with a constant pressure of 1 bar and at various temperatures (100, 140, 180 and 200 K). Then, they were simulated for another 50 ps under the *NPT* ensemble to calculate the DOS by taking the discrete Fourier transform of the mass-weighted VACFs of atoms, specifically given as^69^:


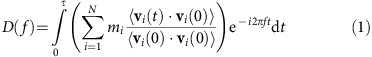


where *f* is the phonon vibration frequency; **v**_*i*_(*t*) and **v**_*i*_(0) are the atomistic velocities of atom *i* at time *t* and 0, which are collected every 10 fs; *m*_*i*_ is the mass of atom *i*; *N* is the total number of atoms belonging to certain species (CH_3_NH_3_^+^ or PbI_3_^−^); *τ*=50 ps is the auto-correlation time period used for data sampling; the angular brackets denote the time-averaged velocity auto-correlation functions. The Savitzky–Golay filter was employed to eliminate the thermal noise (Supplementary Fig. 13).

### Data availability

The data that support the findings of this study are available from the corresponding authors upon reasonable request.

## Additional information

**How to cite this article:** Li, B. *et al*. Polar rotor scattering as atomic-level origin of low mobility and thermal conductivity of perovskite CH_3_NH_3_PbI_3_. *Nat. Commun.* **8,** 16086 doi: 10.1038/ncomms16086 (2017).

**Publisher’s note:** Springer Nature remains neutral with regard to jurisdictional claims in published maps and institutional affiliations.

## Supplementary Material

Supplementary Information

## Figures and Tables

**Table 1:** Comparison of frequencies (in cm^−1^) of typical optical phonon modes.

**No.**	**INS (powder)**^21^	**INS (crystal; this work)**	**DFT (*****P*****1)**^21^	**MD (this work)**
1	18	18.4	21	23
2	−	25.1	−	27
3	30	30.6	28	31
4	−	35.1	−	35
5	40	40.5	38	39
6	53	50	47	48
7	−	57	−	57
8	66	65	58	63
9	−	69	−	69
10	−	73	−	73
11	87	85.5	68	83
12	93	92.8	79	95
13	102	101.6	90	102
14	110	115	106	109
15	118	123	109	120
16	124	131	115	131
17	146	147	130	141
18	−	159	−	153
19	173	167	153	173
20	182	182.3	157	180
21	−	209.7	−	250
22	304	303.3	302	341,352,368

The INS results at 5 K are taken from Fig. 3 while the MD results at 100 K from Supplementary Fig. 6. The numbers correspond to ones appearing the square brackets in these two figures. The data on a powder sample is from ref. 21 along with the associated with DFT calculations on *P*1 model.

**Table 2:** Comparison of properties of acoustic phonons for the tetragonal phase.

**Velocity (m s**^−**1**^**)**		**Mean-free-path (Å)**		**Methods**
**Transverse**	**Longitudinal**	**Transverse**	**Longitudinal**	
970	3,270	−	−	DFT (ref. 36)*
1,445	2,250	19.99	38.19	DFT anharmonic (ref. 35)*
∼1,500	∼2,800	<100		MD (ref. 38)
∼1,200		−		Transport (ref. 37)
1,155	2,841	42	125	INS (this work)

^*^Based on the cubic phase. The specific data of ref. 35 was requested from the authors.

**Table 3:** Timescales of atomic dynamics and related physical properties.

**Phases**	**Relaxation time of rotational modes (ps)**		**Lifetime of phonons (ps)**		***κ*** **(W m**^−**1**^**K**^−**1**^**)**^16^	***μ*** **(cm**^**2**^ **V**^−**1**^ **s**^−**1**^**)**^43^
	***C***_**3**_	***C***_**4**_	**TA**	**TO (12.6 meV)**		
Orthorhombic (150 K)*	23 (1)	Frozen	5.40 (88)	0.62 (7)	∼0.75	∼4.5
Tetragonal (180 K)	0.71 (3)	64 (2)	3.61 (42)	<<0.62	∼0.65	∼2.2

The orientational disorder of dipoles sets in at the transition, where lifetimes of optical phonons and mobility (*μ*) undergo giant changes while acoustic phonons and thermal conductivity (*κ*) are less susceptible.

^*^Relaxation times of rotational modes for orthorhombic phase are taken at 140 K.
