# Supplementary material for: Polar rotor scattering as atomic-level origin of low mobility and thermal conductivity of perovskite CH3NH3PbI3
Source: Nat Commun. 2017 Jun 30;8:16086. doi: 10.1038/ncomms16086 (PMC5497077; doi:10.1038/ncomms16086)
Supplement: Supplementary Information [file ncomms16086-s1.pdf]

Title of file for HTML: Supplementary Information
Description: Supplementary Figures, Supplementary Table and Supplementary References

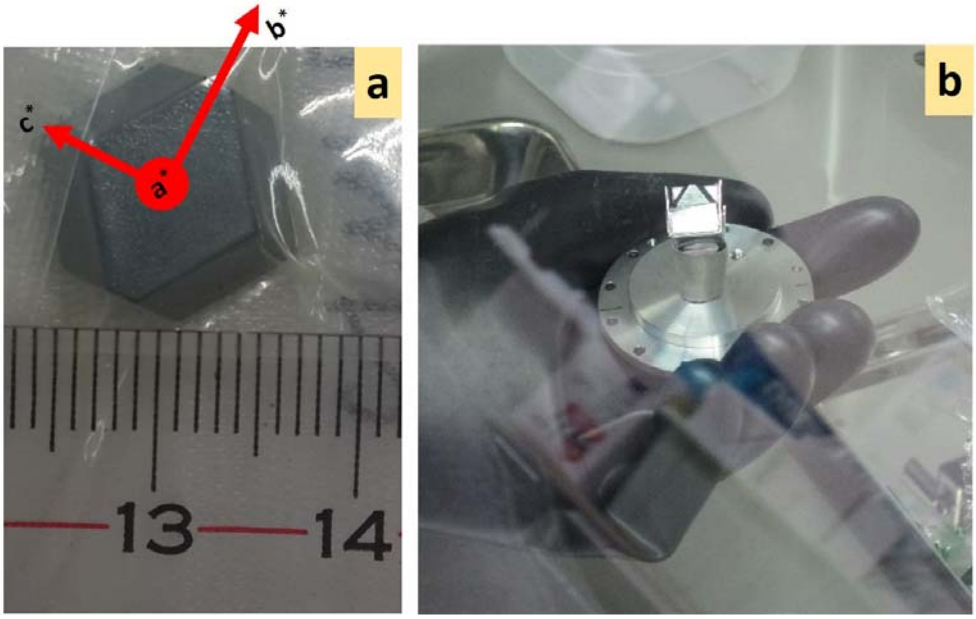

Supplementary Figure 1 | Photographs of the single crystal $CH_3NH_3PbI_3$ used in neutron scattering measurements. (**a**) highlights the dimensions (about 1 cm in length) and the crystallographic directions. (**b**) shows the configuration of the crystal attached on the aluminium sample holder.

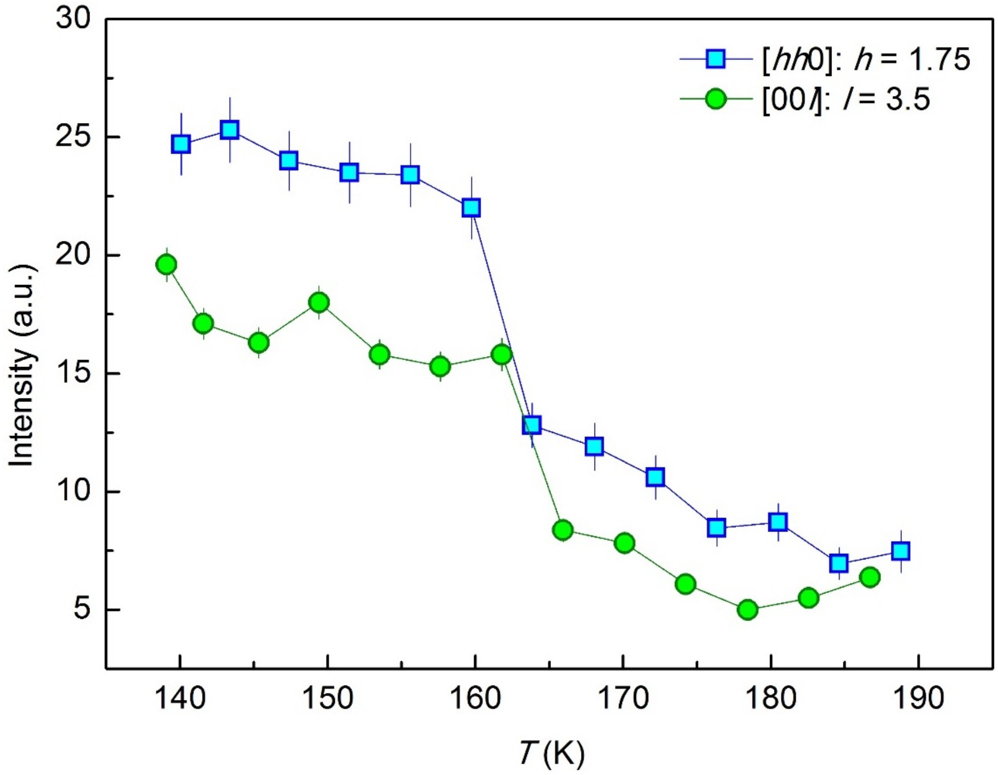

Supplementary Figure 2 | Temperature dependence of incoherent elastic scattering intensity along two directions. The drop of intensity around 165 K is due to the orthorhombic-to-tetragonal phase transition.

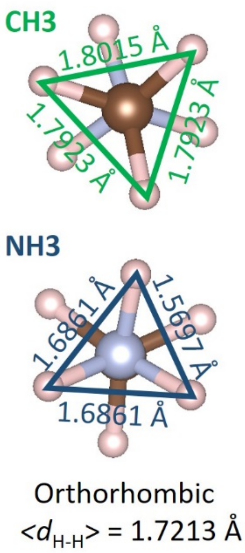

Supplementary Figure 3 | The H-H bond length in the orthorhombic phase. The crystal structural data is obtained from ref. [S1].

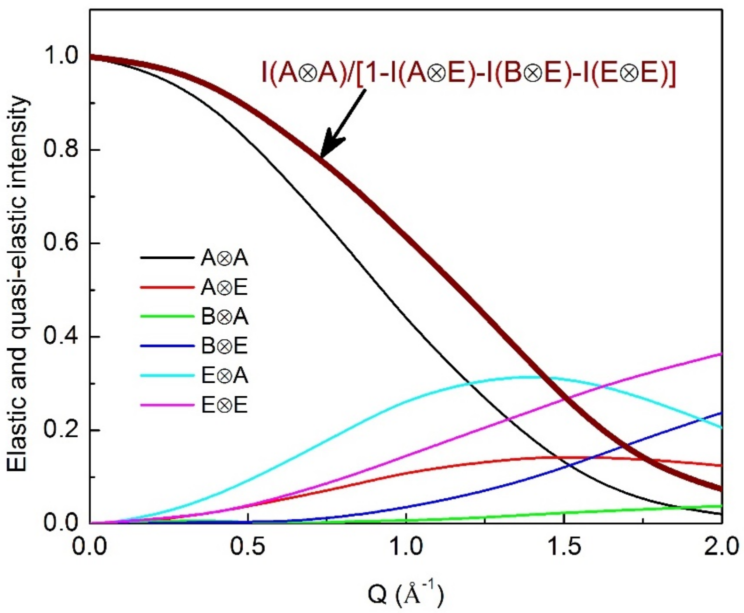

Supplementary Figure 4 | The elastic and quasi-elastic intensity in the $C_4 \otimes C_3$ jumping rotational model of the tetragonal phase[S2]. A⊗A is elastic, i.e., elastic incoherent structure factor (EISF) while others are quasi-elastic intensity. The thick solid line displays the ratio between the elastic intensity and the total intensity excluded the C_3 component. At 180 K, the C_3 mode is much faster than the C_4 mode so that the C_3-mode-related contribution falls into the background in the DNA data. Thus, the calculated EISF is overestimated due to the smaller total intensity missing the C_3-related contribution. This is the reason why the C_3 component is excluded above in the model EISF calculation.

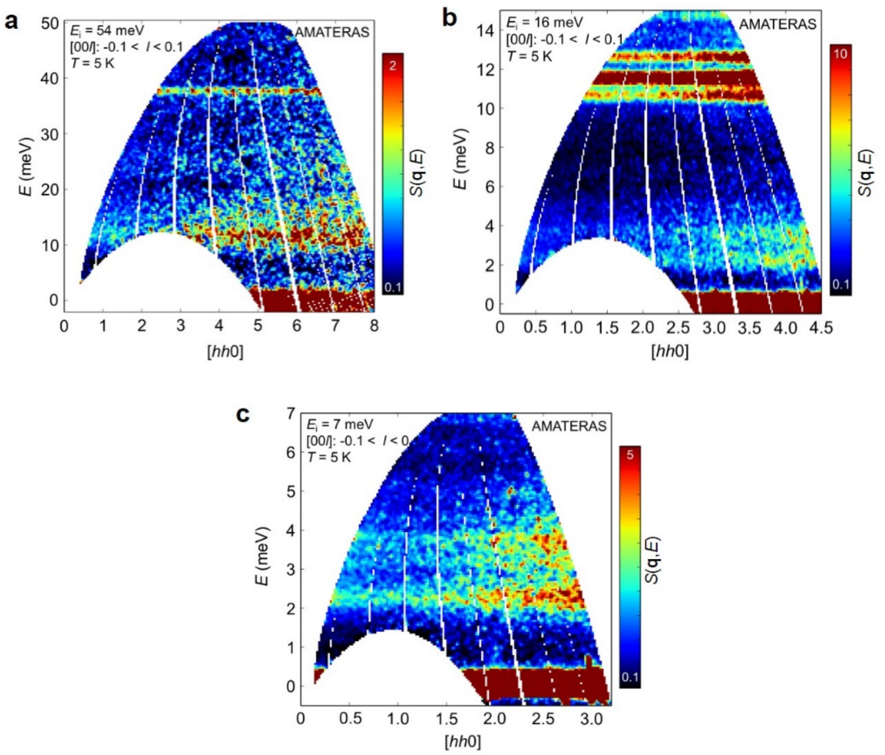

Supplementary Figure 5 | Longitudinal phonon spectra at 5 K along [*hh*0]. (**a**,**b**,**c**) The contour plots of $S(\mathbf{q},E)$ obtained at AMATERAS with E_i of 54, 16, and 7 meV.

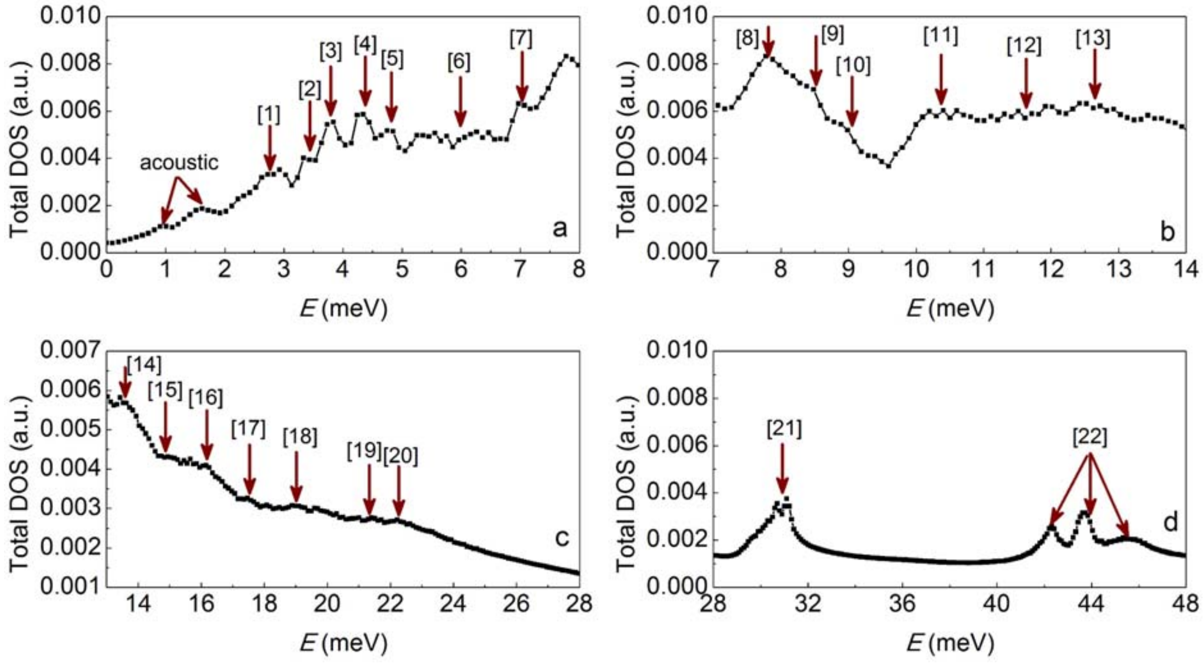

Supplementary Figure 6 | (**a**,**b**,**c**,**d**) **The energies of phonon modes in calculated total phonon density of state (DOS) at 100 K.** The arrows point out the peak positions. The numbers in the square brackets correspond to ones shown in **Fig. 3d**, **e**, **f** and **Table 1**. Note that the horizontal axes of **a**,**b**,**c** are slightly overlapped for clarity.

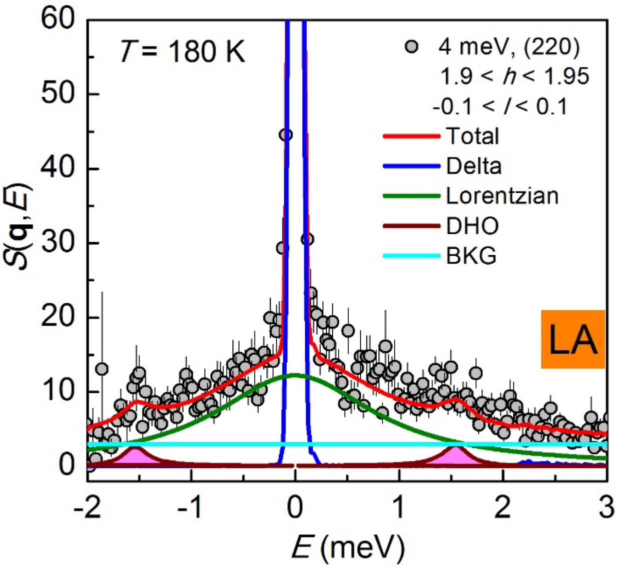

Supplementary Figure 7 | Longitudinal acoustic (LA) phonon spectrum near the (220) zone center at 180 K. Multi-component fitting is shown with solid lines, where the delta function carries the elastic component; the Lorentzian function describes the quasi-elastic component originating from the jumping rotation mode; damped harmonic oscillator (DHO) function accounts for the LA phonon; and the straight line is for background (BKG).

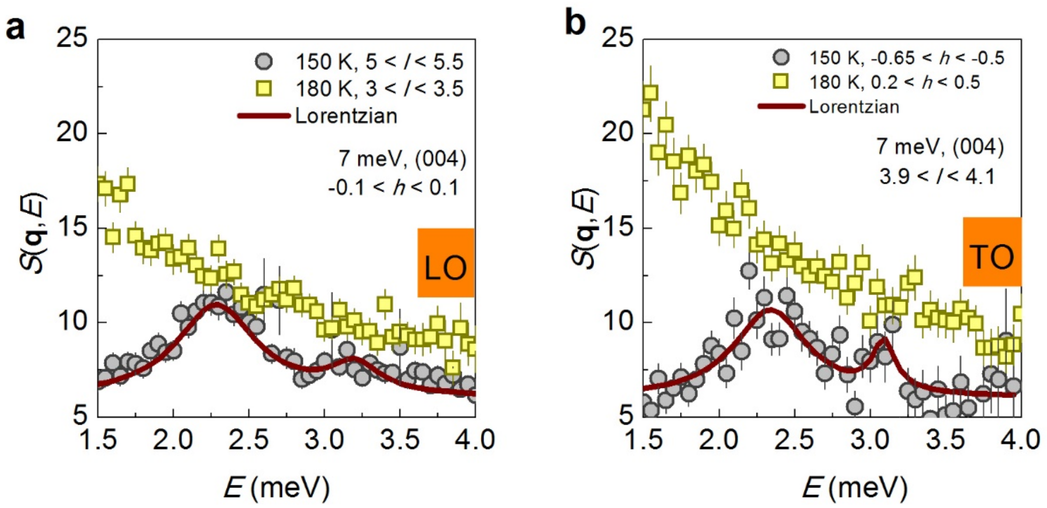

Supplementary Figure 8 | Low-energy optical phonons of the (004) Brillouin zone at 150 and 180 K. Both longitudinal optical (LO) shown in (**a**) and transverse optical (TO) phonons shown in (**b**) are significantly broadened at 180 K. The higher intensity at 180 K is because the C_3 jumping rotational mode become much faster and a stronger quasi-elastic component is imposed.

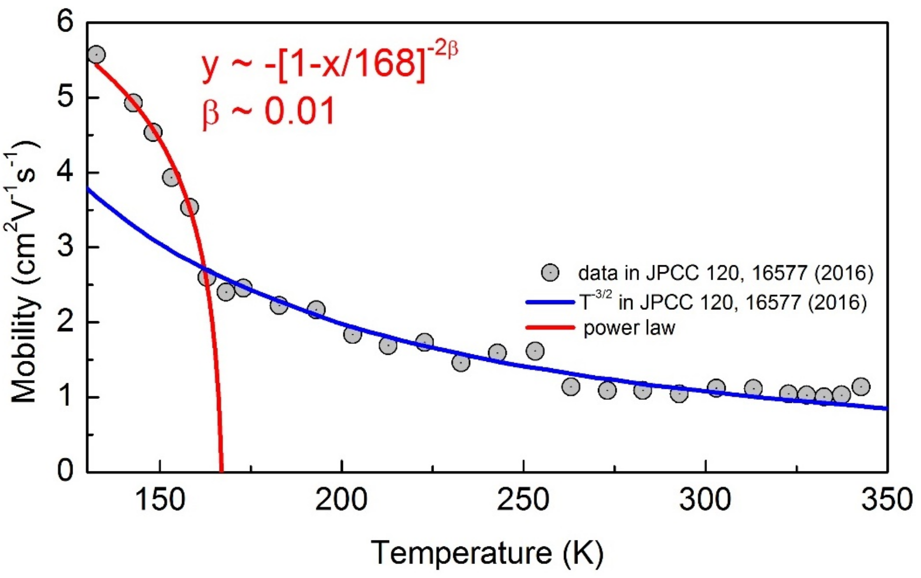

Supplementary Figure 9 | The fitting of mobility to power-law in the low-temperature phase[S3].

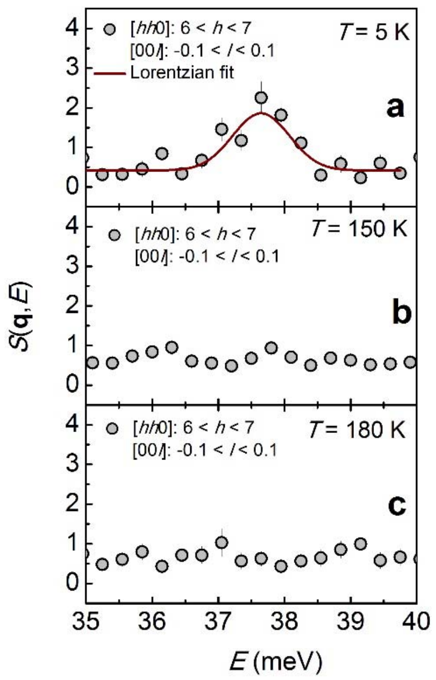

Supplementary Figure 10 | Temperature dependence of the twist mode at 37.6 meV. The momentum-transfer averaged spectra at 5 (**a**), 150 (**b**) and 180 K (**c**), with E_i = 54 meV. The solid lines show the fitting to Lorentzian function. It can be seen that the twist mode is suppressed at 150 K.

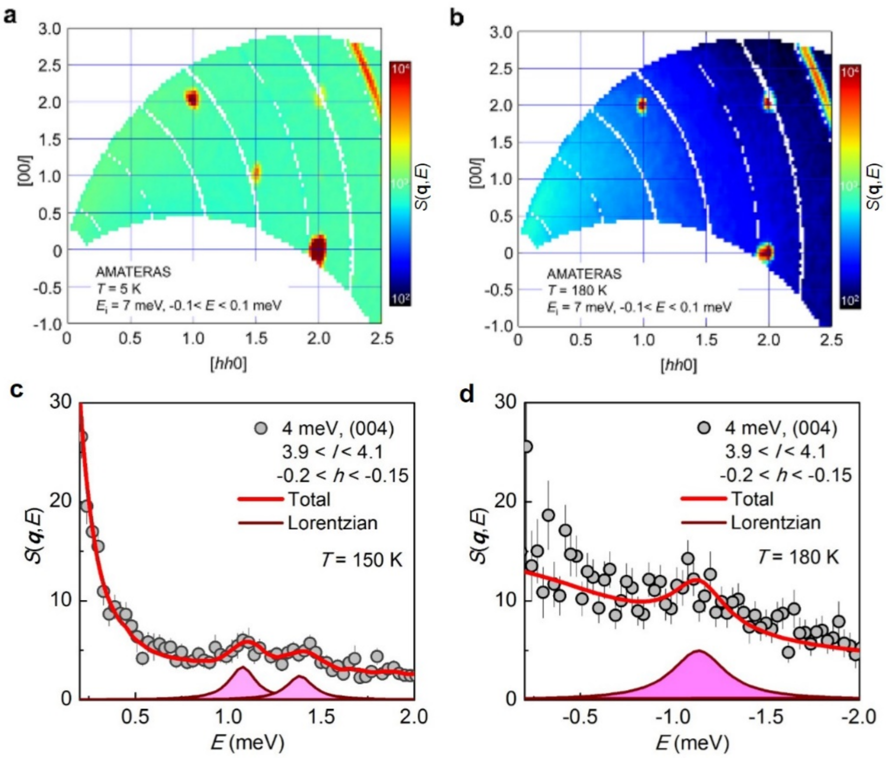

Supplementary Figure 11 | Twinning of the single crystal in the orthorhombic phase. (**a,b**) the elastic cutting of $S(\mathbf{q},E)$ at 5 and 180 K. The spot appearing the $\left(\frac{3}{2}\frac{3}{2}1\right)$ superlattice position is due to the superposition of diffraction spots from two twinned crystals. The red "rings" are Bragg peaks of aluminium used in sample environments while the white "rings" are because of gaps of detector arrays. (**c,d**) The transverse acoustic phonons at 150 and 180 K. The two peaks are found at 150 K due to the twinning, while they are merged at 180 K. Note that we plot the spectrum of negative energy-transfer part in **d**.

Supplementary Table 1 | Summary of instrumental resolutions.

**Condition**	**Resolution (meV)**
**DNA**	0.0036
**AMATERAS: E_i = 4 meV**	0.01
**AMATERAS: E_i = 7 meV**	0.21
**AMATERAS: E_i = 16 meV**	0.60<br>(0.28 at 12 meV)
**AMATERAS: E_i = 54 meV**	2.8<br>(0.76 at 37.6 meV)

These resolution functions were used to fit the quasi-elastic component as well as the acoustic phonons. Specially, as for the optical phonons located at about 12 and 37.6 meV, the inelastic resolution functions are used, given in brackets.

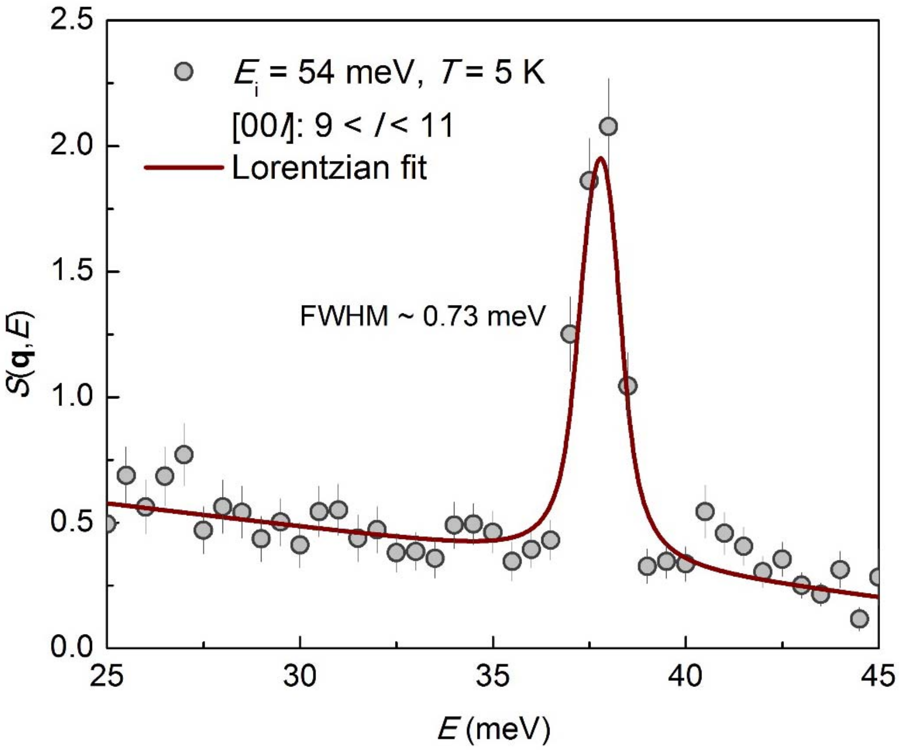

Supplementary Figure 12 | The fitting of the peak of the twist mode at 37.6 meV at 5 K. It can be seen that the full width at half maximum (FWHM) is about 0.73 meV, which is almost identical to the instrumental resolution listed in Supplementary Table 1. This suggests that there is no additional broadening found within our resolution.

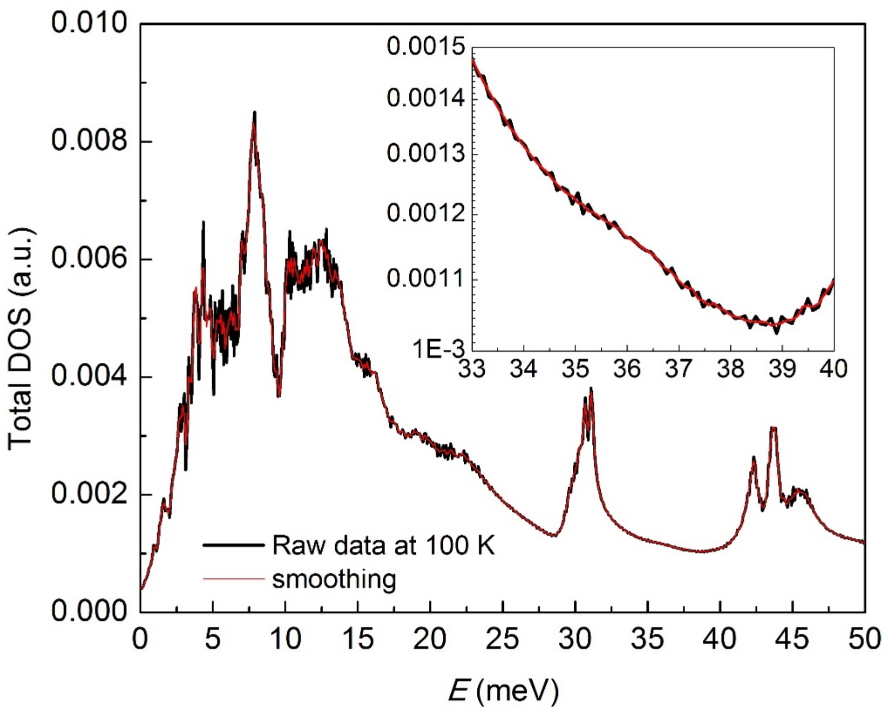

Supplementary Figure 13 | The smoothing of raw data of total phonon density of state (DOS) at 100 K by applying the Savitzky–Golay filter. As highlighted in the inset, the oscillation due to thermal noise is eliminated.

Supplementary References:

[S1]. Weller, M. T. *et al.* Complete structure and cation orientation in the perovskite photovoltaic methylammonium lead iodide between 100 and 352 K. *Chem. Commun.* **51**, 4180–4183 (2015).

[S2]. Chen T. *et al.* Rotational dynamics of organic cations in the $CH_3NH_3PbI_3$ perovskite. *Phys. Chem. Chem. Phys.* **17**, 31278 (2015).

[S3]. Gélvez-Rueda, M. C. *et al.* Effect of cation rotation on charge dynamics in hybrid lead halide perovskites. *J. Phys. Chem. C* **120**, 16577–16585 (2016).
